# Metformin as Host-Directed Therapy for TB Treatment: Scoping Review

**DOI:** 10.3389/fmicb.2020.00435

**Published:** 2020-04-29

**Authors:** Nikita Naicker, Alex Sigal, Kogieleum Naidoo

**Affiliations:** ^1^Centre for the AIDS Programme of Research in South Africa, Durban, South Africa; ^2^Africa Health Research Institute, Durban, South Africa; ^3^School of Laboratory Medicine and Medical Sciences, University of KwaZulu-Natal, Durban, South Africa; ^4^Max Planck Institute for Infection Biology, Berlin, Germany; ^5^MRC-CAPRISA HIV-TB Pathogenesis and Treatment Research Unit, Doris Duke Medical Research Institute, University of KwaZulu-Natal, Durban, South Africa

**Keywords:** tuberculosis, metformin, host-directed therapy, *Mycobacterium tuberculosis*, adjuvant

## Abstract

Tuberculosis (TB) disease is an international health concern caused by the bacteria *Mycobacterium tuberculosis (Mtb)*. Evolution of multi-drug-resistant strains may cause bacterial persistence, rendering existing antibiotics ineffective. Hence, development of new or repurposing of currently approved drugs to fight *Mtb* in combination with existing antibiotics is urgently needed to cure TB which is refractory to current therapy. The shortening of TB therapy and reduction in lung injury can be achieved using adjunctive host-directed therapies. There is a wide range of probable candidates which include numerous agents permitted for the treatment of other diseases. One potential candidate is metformin, a Food and Drug Administration (FDA)-approved drug used to treat type 2 diabetes mellitus (DM). However, there is a scarcity of evidence supporting the biological basis for the effect of metformin as a host-directed therapy for TB. This scoping review summarizes the current body of evidence and outlines scientific gaps that need to be addressed in determining the potential role of metformin as a host-directed therapy.

## Introduction

Tuberculosis (TB), although largely a curable disease, remains the leading cause of mortality globally, fueled by the unprecedented increase in anti-mycobacterial drug resistance. In 2018, the World Health Organization (WHO) reported 10 million people falling ill with active TB (World Health Organization, 2018). Of these, 558,000 were new cases resistant to rifampicin–a key drug in the current first-line treatment. Eighty-two percent of the rifampicin-resistant cases had multi-drug-resistant TB (MDR-TB), which includes, at a minimum, resistance to the first line drug isoniazid (World Health Organization, 2018). While the incidence of drug resistance is approximately 5% of total TB cases, 17% of TB deaths were due to drug-resistant strains (World Health Organization, 2018).

Treatment regimens for TB consist of combinations of anti-mycobacterial drugs aimed at eradicating infection while preventing the development of resistance and recurrent infection. Anti-TB regimens are lengthy and associated with high toxicity, driving poor treatment adherence and development of MDR and extensively drug-resistant (XDR) TB (Shenoi et al., 2009; Calver et al., 2010; Zumla et al., 2013; Millington, 2018). Current treatment regimens are pathogen-targeted strategies, which are able to be severely compromised by the development of drug resistance by the pathogen (Millington, 2018). Resistance in *Mycobacterium tuberculosis* (*Mtb*) has been attributed to the development of resistance-conferring mutations in genes that encode drug targets or drug activating enzymes (Gygli et al., 2017). Another phenomenon also exists which participates in the development of drug resistance. A study by Sharma et al. (2015) showed ion metabolism to play a crucial role in contributing to amikacin and kanamycin resistance (Sharma et al., 2015). Furthermore, proteomics and bioinformatics expression studies of drug-resistant *Mtb* have defined the overexpression of uncharacterized and hypothetical proteins to possibly counteract the effect of anti-TB drugs (Jiang et al., 2006; Sharma et al., 2010; Kumar et al., 2013; Sharma and Bisht, 2017). As a result, further exploitation is necessary for the advancement of novel therapeutic agents which can directly be targeted to a protein and/or a gene accountable for resistance (Sharma et al., 2016, 2017). In addition, antibiotic pressure induces a stress response in *Mtb.* This, in combination with the capability of bacteria to engage into a dormant state, creates a subpopulation of *Mtb* with decreased sensitivity to TB chemotherapy which preferentially targets dividing bacteria (Balaban et al., 2004; Lewis, 2007; Wakamoto et al., 2013; Gygli et al., 2017). Current therapeutic strategies include pretreatment drug susceptibility screening for only one anti-TB drug. Hence, the standard-of-care multidrug treatment regimens comprise drugs of uncertain efficacy.

In 1991, the “World Health Assembly Resolution” identified TB as a “major global health problem,” and several strategies such as the “WHO Directly Observed Treatment, Short-course (DOTS) strategy” and the “Stop TB strategy” have been implemented (Uplekar and Raviglione, 2015). Between 2000 and 2015, an estimated 49 million lives were saved, with a 22% drop in TB deaths (World Health Organization, 2016a). However, only one in five people needing MDR-TB treatment initiated such therapy (World Health Organization, 2016a). In May 2014, a new strategy and targets for TB prevention, the End TB Strategy, were set. The purpose of this strategy is to terminate the international TB epidemic, with the following targets compared to 2015: “90% reduction in number of TB deaths, 90% reduction in TB incidence rate, and 90% reduction in TB affected families facing unfortunate costs due to TB” by 2035 (Uplekar et al., 2015; World Health Organization, 2016b). The currently adopted End TB strategy, while ambitious, can only be realized through novel approaches in treating and preventing TB.

Host-directed therapy (HDT) provides a largely unexploited approach as adjunctive anti-TB therapy. Firstly, HDT may impair *Mtb* replication and survival by disrupting *Mtb* manipulation of macrophage pathways, thus rendering the bacteria more sensitive to host defenses (Hawn et al., 2015). The current search for novel therapeutics has focused on the use of repurposed drugs aimed at optimizing the host’s response against the mycobacterium (Sharma et al., 2017). HDT has been proposed as an adjuvant therapy for TB, to improve the efficacy of current treatment outcomes. One plausible resolution to the challenge of antibiotic resistance and non-replicating bacterial death is targeting the host as opposed to the pathogen (Rayasam and Balganesh, 2015) because it depends neither on bacterial division nor on the bacterial susceptibility to drugs. Metformin (MET) is a Food and Drug Administration (FDA)-approved drug, well-known for its glucose-lowering effect on type 2 diabetes (T2D) individuals (Foretz et al., 2014). A group of studies have reported the potential role of MET as an adjunctive therapy for TB (Singhal et al., 2014; Singhal and De Libero, 2017; Degner et al., 2018). However, relatively little is known on how MET modulates the cellular interaction between *Mtb* and macrophages. We therefore pursued to amalgamate the evidence base on MET as an adjunctive therapy for TB using a scoping study methodology to identify gaps to be attained in future research.

## Methodology

### Scoping Review

The Arksey and O’Malley framework guided this research (Arksey and O’Malley, 2005). The framework involves: (1) finding the research question; (2) finding relevant studies; (3) study choice; (4) recording the data; and (5) organizing, summarizing, and reporting the results.

### Identifying the Research Question

We reviewed existing literature with the aim of exploring what is known about the cellular interaction of MET, specifically defining the role of MET in mediating host–pathogen defense. The primary research question was guided by the subsequent sub-research questions: (1) “What is identified in the literature?”; (2) “What are the research gaps?”; and (3) “What are future research desires?” The results from the scoping review will be used to answer the above questions, to enable a better understanding of the research gaps and to guide future work in determining the potential use of MET as HDT for TB.

### Eligibility of the Research Question

An amended PICOS (Population/Problem, Intervention, Comparison, Outcomes and Study setting) framework was used to identify the suitability of the research question (Table 1). We used the PICOS framework to clearly define a well-focused research question for us to obtain appropriate resources and search for relevant evidence in the literature.

**TABLE 1:** PICOS framework for determination of eligibility of review question.

Criteria	Determinants
Problem	Anecdotal data suggests that MET, a drug used to treat type II diabetes mellitus, affects the ability of macrophages to control intracellular *Mtb*.
Intervention	Does MET affect the ability of macrophages to control intracellular *Mtb.*
Comparison	Absence of MET.
Outcomes	Effect on macrophage phagolysosomal activity.
	Synergistic effect with current TB therapy.
Study setting	Includes studies worldwide.

*MET, metformin; *Mtb*, *Mycobacterium tuberculosis*; PICOS, Population/Problem, Intervention, Comparison, Outcomes and Study setting; TB, tuberculosis.*

### Identifying Relevant Studies

We included primary studies demonstrating clear empiric evidence published in peer-reviewed journals. Qualitative, quantitative, or mixed methods studies that addressed the research question from January 2007 to July 2019 were included. For this scoping review, English electronic academic databases, Google scholar, Ovid, PubMed, Science direct, Scopus, Web of science, and the WHO website, were reviewed. The search phrases included MET as HDT, MET and macrophages, and MET as adjunctive therapy.

### Study Selection

A set of eligibility criteria for studies to be included was developed to warrant consistent study selection applicable to our research question. Studies that met the following inclusion criteria were selected:

Inclusion:

• Focus on MET as a possible HDT agent
• Studies evaluating the cellular interaction of MET with *Mtb* and macrophages
• Published from January 2007 to July 2019
• No language restriction provided that the articles can be translated to English

The following studies were excluded:

• Studies which do not focus on MET as a possible HDT agent
• Studies which do not focus on cellular interaction of MET with *Mtb* and/or macrophages
• Studies published before January 2007
• If the article cannot be translated to English

Relevant data were then extracted from articles that screened successfully.

### Charting the Data

As per the recommendations by Arksey and O’Malley (2005), data were extracted from eligible articles. Recording the data involved consolidating and understanding data as per key themes. A charting data form was generated to ensure that the most suitable variables and themes are included to answer the research question. The data extraction tool on excel was used to confirm applicable and resourceful data extraction of evidence associated with MET and HDT. Recording of the data included the use of excel spreadsheets for data capturing for each article and to ensure the extracted data were comparable among articles.

### Collating, Summarizing, and Reporting of Results

To increase consistency in reporting of results, we used the recommended three-step approach, i.e., exploring the data, writing of results, and applying sense to the results (Levac et al., 2010). Following data collection and abstraction, synopses were produced and collated for reporting purposes. Research gaps were recognized grounded on recommendations from “authors, key findings, gaps, and lack of evident research.”

## Results

### Metformin as an Adjunct to Tuberculosis Treatment

**FIGURE 1:**
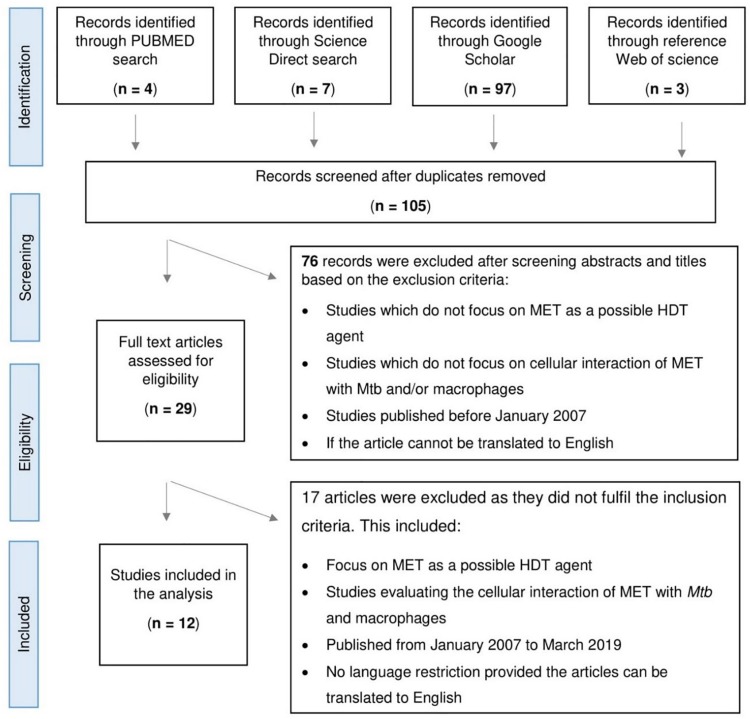
Flowchart depicting the process of article selection for scoping study assessing the role of Metformin (MET) in tuberculosis.

Selected studies included *in vitro*, *in vivo*, and retrospective studies (Figure 1). MET as an adjunctive therapy was demonstrated by Singhal et al. (2014) and Lee et al. (2018) using an *in vitro* model and in a retrospective cohort study, respectively. The *in vitro* model showed MET’s ability to facilitate phagosome–lysosome fusion, reduce chronic inflammation in the lung, enhance immune response, and potentiate the effectiveness of standard TB drugs. In addition, the retrospective cohort demonstrated improved 2 months sputum culture conversion with adjunctive MET treatment, underscoring its potential utility in TB (Singhal et al., 2014; Lee et al., 2018). Conversely, Dutta et al. (2017) provided evidence that contradicted the above results. This group observed MET to have no significant effect on mice treated with MET in combination with the first-line TB regimen (Dutta et al., 2017).

### Effect of Metformin on *M. tuberculosis* Metabolic Pathways and Replication

Evidence showing the effect of MET on bacterial replication was reported in three studies. Authors concluded that MET controlled *Mtb* replication via different methods. Singhal et al. (2014) demonstrated that MET increased mitochondrial reactive species production which led to the growth control of intracellular drug-resistant *Mtb* strains; this facilitated phagosome–lysosome fusion in macrophages. Furthermore, MET promoted accumulation of CD8^+^ and CD4^+^ T cells in the lung autonomously of infection. Antimicrobial activity observed in two *in silico* systems (Vashisht et al., 2014; Vashisht and Brahmachari, 2015) (a) elucidated prospective molecular processes and pathways to circumvent *Mtb* persistence; (b) provided evidence that bacterial NDH-I complex is analogous to mitochondrial complex-I in structure and function (Vashisht et al., 2014; Vashisht and Brahmachari, 2015). Complex-I of the mitochondria is the first enzymatic complex in the respiratory chain, accountable for the generation and transport of electrons forming a proton gradient across the inner membrane of the mitochondria which drives the production of ATP. ATP is the main energy source for metabolic functions. Similarly, bacterial NDH-I is accountable for an assortment of functions such as respiration and cyclic electron flow (Yagi et al., 1998), making NDH-I a potential target to inhibit bacteria.

### Impact of Metformin on Morbidity and Mortality

Two of the 10 studies reported the impact of MET on mortality during TB therapy. Degner et al. (2017) observed a decrease in mortality rate among diabetic TB patients receiving MET concomitantly with TB treatment. Additional findings from this study included reduced likelihood of remaining culture positive at 2 months and improved glycemic control. Degner et al. (2018) also reported reduced mortality rate during TB treatment among patients receiving both MET and TB treatment. Both studies concluded that MET is a potential adjunctive HDT for TB therapy.

### Effect of Metformin on Tuberculosis Outcomes

Studies by Alisjahbana et al. (2007) and Singhal et al. (2014) observed reduced TB disease severity when MET was used with first line TB therapy. Alisjahbana et al. (2007) studied TB patients with and without diabetes mellitus (DM) and showed a greater likelihood of delayed smear and culture conversion and unfavorable outcomes among patients with both TB and T2D. In two retrospective cohort studies, Singhal et al. (2014) showed a statistically significant reduction in TB severity. Improved clinical outcomes and fewer pulmonary cavities were observed among TB patients receiving MET (Singhal et al., 2014). Of note, studies done in different population groups concur with findings of reduced mortality during TB treatment among T2D patients cotreated with MET (Degner et al., 2017, 2018). Benefits of MET were observed by Srujitha et al. (2017) who observed a protective role of MET against TB infection and showed that lack of glycemic control is a hazard for TB incidence. Additional benefits of MET such as reduced rates of TB infection (Lee et al., 2018) and increased sputum culture conversion in those with higher bacillary burden (Degner et al., 2018; Novita et al., 2019) have also been observed.

### Metformin Effect on the Immune Response to *M. tuberculosis*

MET alters the amount and dispersal of circulating immune cells. Lachmandas et al. (2019) observed that following MET exposure, there was a temporary elevation in overall white blood cell and neutrophil counts (increase over 6 days with return to pre-MET treatment baseline by 21 days). The ratio of monocytes, the precursor cells for macrophages, to lymphocytes was increased in peripheral blood (Lachmandas et al., 2019). MET was observed to modulate the host’s innate immune response (Singhal et al., 2014; Lachmandas et al., 2019; Novita et al., 2019), including MET-induced increases in whole-blood reactive oxygen species (ROS) production and strong upregulation of genes responsible for ROS production (Lachmandas et al., 2019). Furthermore, Singhal et al. (2014) showed *in vitro* that MET restricts bacterial growth by increasing the production of ROS. ROS are known to promote naive T cell proliferation and play a critical role in modulating the development and effector functions of various T lymphocyte subsets (Yarosz and Chang, 2018). Arai et al. (2010) identified an underlying mechanism of MET inhibition of tumor necrosis factor (TNF) production and transcription factor expression by the ability of MET to inhibit TNF and tissue factor production with the addition of the stimulant lipopolysaccharide (LPS) or oxidized low-density lipoprotein (Lachmandas et al., 2019). Novita et al. (2019) measured levels of microtubule-associated Protein 1 light chain 3B (MAP1LC3B), superoxide dismutase (SOD), and interferon in a small group of 22 patients with both TB and T2D. This group observed an increased MAP1LC3B, SOD, and interferon before and after MET treatment (Novita et al., 2019). This is important since MAP1LC3B is a key protein representing autophagy; SOD is an important antioxidant that is also thought to enhance the bactericidal effects of isoniazid, and interferon-gamma levels are known to play an important role in autophagy (Novita et al., 2019). In two *in silico* system studies Vashisht et al. identified metabolic mechanisms related to NAD biosynthesis during *Mtb* infection and observed directive detouring of metabolic fluidities via NAD biosynthesis pathway (Vashisht et al., 2014; Vashisht and Brahmachari, 2015). Pathogens possess the ability to hijack enzymes responsible for maintaining the NAD+ homeostasis of the host this leads to a metabolic dysfunction allowing for the survival and persistence of the pathogen (Grady et al., 2012; Imai, 2016; Mesquita et al., 2016; Matalonga et al., 2017). Dissimilar classes of pathogens have been reported to cause fluctuations in NAD+ levels within infected cells. Variations of NAD+ levels are projected to disturb effector functions of immune cells and, subsequently, disturb the clearance or perseverance of infections. NAD+ levels are largely reliant on the enzyme IDO and the “NAD+-consuming enzymes SIRTs, PARPs, and CD38” (Grady et al., 2012; Imai, 2016; Mesquita et al., 2016; Matalonga et al., 2017). These catalysts play imperative roles in “energy metabolism, cell survival, proliferation and effector functions,” rendering these enzymes as probable targets for HDT.

## Discussion

**FIGURE 2:**
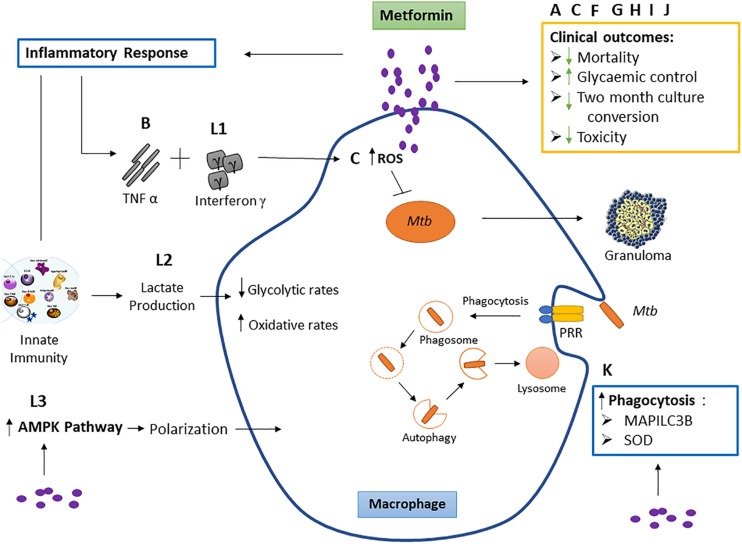
The effect of metformin on macrophage function and clinical outcomes. Studies labeled A, C, F, G, H, I, and J represent the effect of metformin on respective clinical outcomes. These include reduced mortality, 2-month culture conversion and toxicity, and improved glycemic control. Studies B and L: outcome 1 show the effect of metformin on tumor necrosis factor (TNF)-α and interferon γ. TNF-α acts together with interferon γ, causing the production of reactive nitrogen intermediates and facilitating the tuberculostatic function of macrophages and the migration of immune cells to the infection site, contributing to granuloma formation. Study C shows metformin to increase macrophage reactive oxygen species (ROS) production. Evidence suggests that macrophage-produced ROS is responsible for increasing macrophage microbicidal activity by directly killing bacteria (Tan et al., 2016). This study is not part of the inclusion criteria. Study K observes metformin increases superoxide dismutase (SOD) and microtubule-associated proteins 1A/1B light chain 3B (MAP1LC3B). *Mycobacterium tuberculosis (Mtb)* binds the pattern recognition receptor (PRR) on the macrophage which initiates phagocytosis. Superoxide dismutase (SOD) (produced as a by-product of oxygen metabolism within cells) enables clearance of bacteria and restricts inflammation in response to infection by encouraging bacterial phagocytosis, and MAP1LC3B is representative of autophagy while SOD induces autophagy. Study L: outcome 2 shows metformin to increase lactate production within cells. Lactate is formed in large quantities by innate immune cells during inflammatory activation. Lactate modulates the immune cell metabolism which translates to decreased inflammation and ultimately functions as a negative feedback signal to avoid unwarranted inflammatory responses. Study L: outcome 3 observed a macrophage-targeting mechanism for the anti-inflammatory effects of metformin via polarization.

Reviewed published literature supports a biologic basis for the use of MET as HDT for *Mtb* infection (Alisjahbana et al., 2007). Evidence supporting MET use as adjunctive therapy for TB was observed *in vitro, in vivo*, in retrospective, and in modeling studies. Taken collectively, this review suggests that MET has the potential to improve existing shortfalls of current TB therapy (Figure 2). Metformin was first approved by the US FDA in 1994 for DM and insulin resistance and is incorporated within the “WHO’s List of Essential Medicines,” as it is regarded among the most effective and safe medicines needed within health systems (Crofford, 1995; World Health Organization, 2013). Experiments conducted in preclinical models aimed at assessing the biologic efficacy of MET in TB remain warranted, as repurposing of MET for TB is being explored (World Health Organization, 2013). It is well-established that HDT agents produce effects on multiple host targets both related and unrelated to a specific pathway. Hence, HDT studies in TB need to first establish the mechanism, i.e., the specific molecular and/or cellular target within specific host immune response pathway for TB. The development of MET as a potential HDT for TB can be accelerated by identifying biomarkers that specify immediate effects on these targets *in vivo*. Despite the absence of HDT-specific biomarkers, traditional treatment response markers such as clinical response, radiologic improvement, and microbiologic clearance of TB remain potential surrogate markers of anti-TB HDT efficacy.

The evaluation of MET during 2-month culture conversion, illness, and death exhibited that diabetes independently presents an amplified risk of death during TB therapy (Alisjahbana et al., 2007; Degner et al., 2017, 2018; Srujitha et al., 2017). Age, cancer, and chronic kidney disease are associated with increased odds of mortality and morbidity during TB treatment (Alisjahbana et al., 2007; Degner et al., 2017, 2018). Upon secondary analysis, the odds of mortality were decreased with concurrent MET use among T2D patients; this was accompanied by a “positive correlation” of only one and/or interim exposure of MET to extended “immunity against TB infection” (Degner et al., 2018). MET was also safe for T2D-TB patients showing no increase in lactate levels, suggesting a potential adjunctive role of MET for TB-DM comorbidity.

Direct modulation of the host immune response by TB HDT agents may change the relationship between infection and swelling by changing the anticipating importance of the host-directed response in TB disease. Thus, advancement of TB HDT agents may require interchange evaluations of irritation, tissue harm, defensive invulnerability, and general antimicrobial adequacy. Compelling evidence using MET as an adjunctive agent to a single anti-tubercular drug showed restricted intracellular growth of *Mtb* within the human macrophage cell line (Singhal et al., 2014). In this study, MET was found to mediate intracellular phagosome–lysosome fusion and induce autophagy of *Mtb*-infected macrophages. Interestingly, blockade of these two mechanisms did not alter MET-mediated inhibition of bacterial growth (Singhal et al., 2014). Additional studies also show that in the presence of MET, there is evidence of increased macrophage autophagy leading to killing or containment of *Mtb*, evidence of control of inflammation seen by elevated TNF-α, interferon-γ, and interleukin-β and initiation of innate and adaptive responses within the host (Arai et al., 2010; Lachmandas et al., 2019). Extreme pro-inflammatory reactions are troublesome as they bring about broad tissue harm before the improvement of *Mtb*-specific adaptive immunity (Singhal et al., 2014). Adjunctive treatment with MET was found to lessen the tissue pathology and to advance immune responses (Singhal et al., 2014). *In silico* analysis provides potential insights into the antimicrobial activity of MET. These analyses found direct “re-routing of metabolic fluxes” via “NAD biosynthesis pathway” and NDH-1 in *Mtb*, thereby presenting a probable alternate mechanism for ATP production, supporting persistence of bacilli (Vashisht et al., 2014). Interestingly, utilizing the mechanical and functional comparisons between NDH-1 of the bacteria and complex-1 of the mitochondria, accompanied by its effect on the host complex-1 supports MET’s role in also inhibiting *Mtb* NDH-1, suggesting a potential mechanism of impact of MET on *Mtb* persisters (Vashisht and Brahmachari, 2015). These studies all suggest potential effectiveness of MET as HDT for TB and underscore the need for further research aimed at establishing how MET elicits these responses.

Pilot preclinical and observational research recommends the application of MET as an adjunctive, host-directed agent for TB therapy. As previously shown in a retrospective analysis of 220 individuals with DM and TB, MET dosed at 250 mg/kg enhanced the efficacy of conventional anti-TB drugs (Singhal et al., 2014). This was also related to reduced TB severity and enhanced clinical outcomes (Singhal et al., 2014). Another retrospective study showed MET to be related to a 3.9-fold decrease in TB incidence with DM patients (Srujitha et al., 2017). Following these deductions, this preclinical study theorized that MET adjunct therapy would supplement the “bactericidal and sterilizing” activities of conventional therapy (Cohn et al., 1990; Ahmad et al., 2010) against chronic TB infection in mice and reduce the period of therapeutic treatment evaluated by decreased microbiological decline (Dutta et al., 2017). However, the study found that MET did not demonstrate activity in mice with chronic TB infection. During therapy, mice treated with MET showed no obvious toxicity (Dutta et al., 2017), but a trend toward enhanced bactericidal activity in the MET group was observed through the “continuation phase” of treatment (Dutta et al., 2017).

Several favorable conclusions have emerged from clinical and experimental studies that suggest improved TB treatment outcomes with concurrent MET use (Tables 2, 3). TB disease progression is extremely influenced by the *Mtb*–host interaction, thus the modulation of cellular host defenses may have a significant contribution in containment of *Mtb* infection. Modulation of biological procedures such as “inflammatory responses, signaling, metabolism or cellular procedures” like autophagy have arose as hopeful targets for HDT. To define the definitive role of MET as adjunctive TB therapy, an assessment of the effect of MET on the ability of macrophages to control intracellular *Mtb* needs to be established. This needs to be supported by further assessing the additive effect of MET on the ability of macrophages to control intracellular *Mtb* in the presence of TB therapy. Major implications of MET HDT would assist in shortening TB treatment, improving TB treatment outcomes and early host eradication of TB infection with potential to reduce TB transmission. All the studies and/or trials published so far are retrospective in nature. The establishment of a role for MET HDT in TB control would initiate a well-designed prospective randomized human study in well-defined populations.

**TABLE 2:** *In vitro*, *in vivo*, prospective, and retrospective studies evaluating the role of metformin in TB: 2007–2019.

Author, Year and journal	Title	Type of study (Country, sample size)	Main objective	Key finding
Alisjahbana et al., 2007, Clinical Infectious Disease	Effect of type 2 diabetes mellitus on presentation and treatment response of pulmonary tuberculosis	Prospective cohort (Indonesia, *n* = 634)	Investigated clinical characteristics and outcomes in TB patients with and without DM	– T2D associated with: (a) more symptoms but not increased severity of TB (b) negative outcomes following anti-TB treatment – Possible pharmacokinetic interaction between TB therapy and oral hypoglycemic agents
Arai et al., 2010, Journal of Pharmacology and Experimental Therapeutics	Metformin, an antidiabetic agent, suppresses the production of tumor necrosis factor and tissue factor by inhibiting early growth response factor-1 expression in human monocytes *in vitro*	*In vitro* study (mononuclear cells from healthy volunteers)	To identify underlying mechanisms of MET inhibition of tumor necrosis factor (TNF) production and tissue factor (TF) expression in human monocytes, stimulated with lipopolysaccharide (LPS) or oxidized low-density lipoprotein (oxLDL)	– MET (10 μM) halted TNF and tissue factor production when stimulated with LPS or oxLDL (*p* < 0.01) – NF-κB, AP-1, and Egr-1 (*p* < 0.01) regulate monocyte production of TNF and TF – The inhibitory effect of MET on TNF and TF production not mediated through inhibition of NF-κB pathway or inhibition AP-1 activation – MET inhibited LPS- or oxLDL-induced phosphorylation of Egr-1
Singhal et al., 2014, Science Translational Medicine	Metformin as adjunct anti-tuberculosis therapy	*In vitro* (Human monocytic cell line – THP-1) *In vivo* (C57BL/6 mice) Retrospective study of two independent cohorts (TB and diabetic)	To determine if MET can be used as an adjuvant with TB therapy	– *In vitro*, MET restricts bacterial growth by increasing production of mitochondrial reactive oxygen species (*p* < 0.0047) –*In vivo*: (a) MET (500 mg/kg) decreased bacillary count in lung and spleen (*p* < 0.001) (b) MET enhanced efficacy of INH – showed by reduced bacillary count in mice lungs co-treated with INH + MET compared to mice receiving INH only (*p* < 0.05) (c) MET (250 mg/kg or 500 mg/kg), MET + INH (10 mg/kg) reduced organ size and tissue lesions – Retrospective cohort 1: (a) MET therapy reduced TB severity and improved clinical outcome (*p* < 0.001) (b) 109 patients treated with MET had fewer pulmonary cavities (*p* < 0.041) – Retrospective cohort 2: (a) MET treatment was associated with reduced T-SPOT reactivity (*p* < 0.05)
Vashisht et al., 2014, Journal of Translational Medicine	Systems level mapping of metabolic complexity in Mycobacterium tuberculosis to identify high-value drug targets	*In silico* (dynamical mathematical models to understand function of biological systems)	Investigated metabolic mechanisms in Mtb, in the presence of TB therapy that potentiate formation of persister phenotypes	– Identified critical proteins for growth and survival of *Mtb* – Formulated novel idea of metabolic persister genes - associated predictions with published *in vitro* and *in vivo* experimental evidence: (a) NAD biosynthesis results in bacterial persistence in *Mtb* during metabolic stress induced by anti-TB treatment (b) Suggest persister genes as possible drug targets to advance their effectiveness and competence of existing antibiotics for drug tolerant bacteria
Vashisht and Brahmachari, 2015, Journal of Translational Medicine	Metformin as a potential combination therapy with existing front-line antibiotics for Tuberculosis	*In silico*	Assessed if MET can be a potential drug candidate for targeting drug tolerant Mtb	– Identified direct re-routing of metabolic fluxes via NAD biosynthesis pathway and respiratory chain complex – I in *Mtb*: (a) Probable alternate mechanism of ATP generation may facilitate persister phenotype formation, demonstrating antibiotic tolerance (b) Targeting proteins encoding for NDH-I and NAD pathway together with front-line antibiotics provides a strategy to counter drug tolerance (c) Development of new therapeutic intervention for TB therapy (d) MET inhibits both bacterial NDH-1 complex and human mitochondria complex-1
Srujitha et al., 2017, The Brazilian Journal of Infectious Diseases	Protective effect of metformin against tuberculosis infections in diabetic patients: an observational study of South Indian tertiary healthcare facility	Observational study (South Indian diabetics with TB *n* = 152 and without TB *n* = 299)	To determine the protective effect of MET against TB in T2D patients To investigate the relationship between poor glycemic control and TB	– Poor glycemic control (HbA1c > 8) observed in experimental (51.7%) vs. control groups (31.4%) – HbA1c < 7 associated with TB protection – 3.9-fold protection against TB with MET in diabetics
Degner et al., 2017, American Thoracic Society	The Effect of Diabetes and Comorbidities on Tuberculosis Treatment Outcomes	Retrospective cohort study (Patients > 13 years with culture confirmed drug-susceptible pulmonary TB, undergoing treatment)	To assess the effect of DM and poor glycemic control on mortality during TB treatment and 2-month TB sputum culture conversion	– TB associated mortality in DM and poor glycemic control (23.6%) vs. mortality in DM and good glycemic control (10.9%) (*p* < 0.001) – 1.9 times greater odds of death during TB therapy than non-diabetic patients (CI 1,37–2.66, *p* < 0.01) – 1.7 times greater odds of remaining culture positive at 2 months in DM vs. non-DM (CI 1.16–3.69, *p* < 0.01) – Diabetic patients had a significant association between MET use and decreased death during TB therapy (CI 0.10–0.65, *p* < 0.01)
Dutta et al., 2017, Antimicrobial Agents Chemotherapy	Metformin Adjunctive Therapy Does Not Improve the Sterilizing Activity of the First-Line Antitubercular Regimen in Mice	*In vivo* (BALB/c mice)	To investigate bactericidal and sterilizing activities of human-like exposures of MET when given in combination with the first-line regimen against TB	– 53.3%, 20%, and 6.6% of mice treated with conventional TB therapy only reverted after therapy at 3.5, 4.5, and 5.5 months, respectively – MET adjunct treatment did not significantly alter reversion proportions, as 46.6% (p = 0.52), 20% (p = 1.0), and 0% (p = 1.0) of mice reverted following therapy for 3.5, 4.5, and 5.5 months, respectively – Mice treated with MET showed no obvious signs of toxicity during treatment period
Degner et al., 2018, Clinical Infectious Diseases	Metformin Use Reverses the Increased Mortality Associated with Diabetes Mellitus During Tuberculosis Treatment	Retrospective cohort study (patients aged ≥ 13 years undergoing treatment for culture-confirmed, drug-susceptible pulmonary TB, *n* = 2416)	(1) To assess the effect of DM on all-cause mortality during TB treatment and 2- and 6-month TB sputum-culture conversion rates (2) To evaluate the effect of metformin use on survival during TB treatment	– 2416 patients undergoing TB therapy were adjusted for age, sex, chronic kidney disease, cancer, hepatitis C, tobacco use, cavitary disease, and treatment adherence – During TB treatment: (a) 29.0% of patients with DM and 13.7% patients without DM experienced the primary clinical outcome, death (*p* < 0.01) (b) 23.9% of patients with DM and 14.2% patients without DM had a 2-month sputum culture positive for *Mtb* (*p* < 0.01) (c) No difference in 6-month TB sputum culture positivity between DM (0.3%) and non-DM (0.8%) patients (p < 0.39) (d) Overall death was 17.6% among metformin users and 31.3% among non-metformin users (hazard ratio, 0.56 [95% CI, 0.39–0.82]) (e) Survival was significantly higher in the metformin group in a log-rank test of Kaplan–Meier survival distributions (*p* < 0.01)
Lee et al., 2018, Korean Journal of Internal Med	The effect of metformin on culture conversion in tuberculosis patients with diabetes mellitus	Retrospective cohort study (patients with culture-positive pulmonary TB diagnosed between 2011 and 2012)	To examine the anti-TB treatment effects of metformin on sputum *Mtb* culture conversion after 2 months of TB treatment	– Baseline characteristics, except for chronic renal disease, were not significantly different between the groups – MET treatment had no significant effect on sputum culture conversion (*p* = 0.60) and recurrence within 1 year after TB treatment completion (*p* = 0.39) – MET improved sputum culture conversion rate in patients with cavitary pulmonary TB, who have higher bacterial loads (odds ratio, 10.8; 95% confidence interval, 1.22 to 95.63) – drug resistance was significantly higher in patients with failure to achieve conversion (10.1% vs. 36.1%, *p* < 0.01) – Patients with cavitary pulmonary TB, experienced higher 2-month culture conversion rates in the MET group compared to the non-MET group (OR, 10.80; 95% CI, 1.22 to 95.63; *p* = 0.03)
Novita et al., 2019, Indian journal of tuberculosis	Metformin induced autophagy in diabetes mellitus – Tuberculosis co-infection patients: A case study	Observational clinical study (T2D patients newly diagnosed with TB)	To measure the levels of microtubule-associated Protein 1 light chain 3B (MAP1LC3B) (autophagy associated), superoxide dismutase (SOD), interferon and interleukin-10, and smear reversion in DM-TB co-infected patients	– All patients in the MET group had sputum smear reversion after 2 months of intensive phase TB therapy – Increases in MAP1LC3B, SOD, and interferon before and after the observation period were significant following MET treatment (*p* < 0.005) – During the intensive period of anti-TB therapy MAP1LC3B and interferon displayed significant changes (*p* < 0.005), and SOD showed no significant changes – MAP1LC3B is representative of autophagy whilst SOD induces autophagy – Interferon is responsible for protecting against TB infection
Lachmandas et al., 2019, The journal of infectious diseases	Metformin alters human host responses to Mycobacterium tuberculosis in healthy subjects	*In vitro* (human monocytes)	To investigate the effects of MET on mTOR signaling, p38 and protein kinase B in non-diabetic individuals	Outcome 1 (L1) – MET significantly decreased *Mtb* lysate–induced cytokine production (*p* < 0.05) – MET inhibited mTOR (strongly influences cellular growth and cytokine production) (*p* < 0.01) causing decreased cellular growth (*p* < 0.01) and cytokine production (*p* < 0.05) Outcome 2 (L2) – MET increased lactate production and glucose consumption (*p* < 0.01) in *Mtb* lysate–stimulated PBMCs from healthy individuals Outcome 3 (L3) – p-AMPK was increased in both unstimulated and *Mtb* lysate–stimulated PBMCs after MET intake – MET increased phagocytosis of *Mtb* in macrophages – In response to the innate host responses to *Mtb –* MET has beneficial effects (regulates inflammatory cytokine messenger RNA stability, cell proliferation, and apoptosis) on cellular metabolism and immune function
Novita et al., 2018, Indian Journal of Tuberculosis	A case risk study of lactic acidosis risk by metformin use in type 2 diabetes mellitus tuberculosis coinfection patients	Observational clinical study Type 2 DM newly TB coinfection outpatients Surabaya Paru Hospital	This study aimed to understand the effect of MET as an adjuvant therapy in TB and insulin simultaneous therapy	– Among 42 participants showed no case of lactic acidosis – No evidence that MET therapy induced lactic acidosis event nor that it increased lactate blood level among individuals with TB pulmonary disease – MET use in type 2 DM TB co-infection did not induce lactic acidosis – Contributes to our understanding on the clinical effect of MET use in type 2 DM TB co-infection.

*DM, diabetes mellitus; MET, metformin; *Mtb, Mycobacterium tuberculosis*; TB, tuberculosis.*

**TABLE 3:** Proposed strengths and limitations of MET as HDT for TB therapy.

Strengths	Limitations
Suitable for Phase II clinical trials	Lack of biological plausibility
Known toxicity profile and cheap	Limited knowledge on cellular interactions in the presence of anti-TB therapy
Potential to shorten the standard anti-TB regimen	Limited knowledge on the interaction with resistant TB strains
Decreased risk of TB in patients with diabetes mellitus	
Potential to limit TB mortality	
Increased probability of 2 months sputum culture conversion	
Enhancing macrophage effector mechanisms	
Decreasing inflammation and/or averting lung damage	

*HDT, host-directed therapy; MET, metformin; TB, tuberculosis.*

## Author Contributions

NN developed the research question, collected and analyzed the data, and compiled the review. KN and AS provided comments and alterations to the study design and proofread the review.

## Conflict of Interest

The authors declare that the research was conducted in the absence of any commercial or financial relationships that could be construed as a potential conflict of interest.
